# Peer Support Targeting the Second Victim Phenomenon: Implementation and Outcomes

**DOI:** 10.7759/cureus.78854

**Published:** 2025-02-11

**Authors:** Amber E High, Sharron Forest

**Affiliations:** 1 Anesthesiology, University of Texas Medical Branch, Galveston, USA; 2 Nursing, University of Texas Medical Branch, Galveston, USA

**Keywords:** adverse events, burnout, mental health services, peer support, psychological safety, safety, second victim, suicide prevention, well-being, workplace resilience

## Abstract

The second victim phenomenon profoundly impacts clinicians and compromises care. Peer support is a preferred and practical resource, yet accounts of successful initiatives are scarce. Our findings revealed an urgent need: 20% of clinicians experienced emotionally distressing work events in the past month, 88% observed affected colleagues, but only 20% felt adequate support was available. In response, we quickly launched a scalable, evidence-based peer support program with peer-to-peer and online resources to safeguard clinician well-being. Over three months, trained peers facilitated 33 support encounters related to adverse events and personal issues, including bullying. An online platform designed to destigmatize mental health and normalize second victimhood received over 100 visits. Post-implementation, 77% reported timely, adequate support, and awareness of the term "second victim" doubled. This cost-effective, rapid rollout enhanced the perceptions of support and fostered a caring culture among clinicians. Our transferable approach offers a proactive solution for all healthcare disciplines to support second victims, mitigate burnout, and enhance suicide prevention efforts.

## Introduction

When a healthcare worker experiences personal or professional negative effects after an adverse or patient safety-related event, it is known as the second victim phenomenon (SVP) [1,2]. Symptoms of SVP can include sleep disturbances, depression, fatigue, guilt, isolation, decreased job satisfaction, discomfort in the work environment, and feelings of despair, all of which can impact performance, jeopardize subsequent patient care, and, in rare but devastating cases, lead to suicide [3,4]. All healthcare workers are vulnerable to SVP, and nearly half of them may experience its potentially damaging effects at some point in their careers [5]. Without recognition and emotional support, clinicians may suffer in silence; feelings of isolation and anguish can spiral into psychological and physical consequences [1,6,7].

The Joint Commission (TJC) has recognized the seriousness of SVP and issued a "Quick Safety" report urging organizations to facilitate immediate peer-to-peer support following adverse events [5]. Access to timely and effective peer support is necessary for healthy coping [6,8]. National leaders, including Congress; the Surgeon General; the National Academies of Sciences, Engineering, and Medicine; the Institute for Health Improvement; the American Hospital Association; the American Society of Anesthesiologists; and TJC, recommend the initiation of evidence-based peer support to promote clinician mental health and well-being, target burnout, and prevent suicide [5,9-16]. Peer support initiatives foster a just culture and psychological safety, contributing to a healthier work environment and improved healthcare outcomes.

In this paper, we describe the implementation of evidence-informed strategies to provide emotional peer support and foster clinician resilience in a department that services five campuses within a large academic health system in Southeast Texas. The project aim was to have at least five anesthesia clinicians in need of support participate in the formal peer support processes each week from September 26, 2022, to December 19, 2022.

Available knowledge

The COVID-19 pandemic spotlighted the critical need for emotional support among healthcare professionals, driving an unprecedented demand for resources to foster mental well-being and resilience [12-14]. The importance of emotional support for healthcare professionals, particularly those experiencing SVP, is increasingly recognized worldwide. An integrative review by Neft et al. [7] concluded that formal peer support programs are healthcare professionals’ most preferred and practical form of support. Quantitative data on peer support effectiveness remains limited. Common challenges in peer support programs include confidentiality, documentation, program awareness, funding, and time constraints. The execution of formal programs has traditionally taken months to years; however, amid the demands of COVID-19, there is an increased emphasis on expediting program initiation rather than waiting. To address funding barriers, experts have recommended building intradepartmental peer support processes as a cost-effective solution [4].

Awareness of a standardized emotional support process alone can have stress-buffering benefits and is associated with improved patient safety culture scores [17]. Investing in emotional support is crucial for leaders who prioritize a just and safe culture, as it promotes individual coping and long-term system-wide stability [18-20]. To enhance the effectiveness of peer support strategies, experts suggest local customization and standardization to promote awareness, engagement, and communication [18,21].

Various toolkits and implementation guides are available to facilitate the training of support teams and have been successfully utilized by healthcare systems nationwide [22-25]. Experts consistently emphasize the importance of open, judgment-free communication among peers and the value of debriefings after critical events to encourage resilience and growth [18,19,21]. Furthermore, identifying key events that warrant support outreach and focusing on the emotional impact rather than event details are recommended to enhance the effectiveness of peer support teams.

## Materials and methods

Context

The Department of Anesthesiology at a large academic hospital system in South Texas includes 35 certified registered nurse anesthetists (CRNAs), 60 physician anesthesiologists, and 65 anesthesia residents. The anesthesia team provides state-of-the-art care throughout five campuses, with clinical responsibilities at approximately 50 individual anesthetizing sites daily for diverse patient populations.

Rationale

Havelock’s Model of Change

Havelock's guide is for any change agent who identifies and desires to understand a problem and the surrounding culture to discover and implement novel solutions [22]. Havelock's Model of Change contains seven aspects in a cycle, beginning with 0, referred to as Care. Care is the initial of consideration of the necessity for action. The action phases include building relationships, communicating relevance, and identifying solutions. These are followed by choosing and implementing the path forward. The final phase ensures the innovation is integrated, monitored, and periodically renewed within the system. Havelock's model helps frame change in groups related to culture. Table 1 describes concepts from Havelock's model [22] and complementary project steps.

**Table 1:** Havelock's model applied to project planning EAP: Employee Assistance Program

Concept	Concept ideas	Application
0-care	Who cares, about what?	Identify problem. Anesthesia clinicians express concern and act as change agents.
1-relate	Whose concern is it?	Raise awareness with stakeholders: anesthesia providers, dept. leaders, admin, chaplains, and EAP. Survey for perceived support, understanding of second victim, safety culture, and solutions.
2-examine	Problem solvable?	Recommend immediate access to peer support at the local level.
3-acquire	Solutions?	Review the evidence, best practices, strategies, toolkits, and networking.
4-try	Best solution?	Identify champions and create a plan customized to the dept. and the culture. Activate interventions.
5-extend	Translate into action.	Widen the circle of users; promote the novel process.
6-renew	Sustain and stay relevant.	Obtain feedback, evaluate, and tailor (this step is iterative). Stay current with literature. Add team champions. Expand to other units.

Scott’s Three-Tiered Model

**Figure 1:**
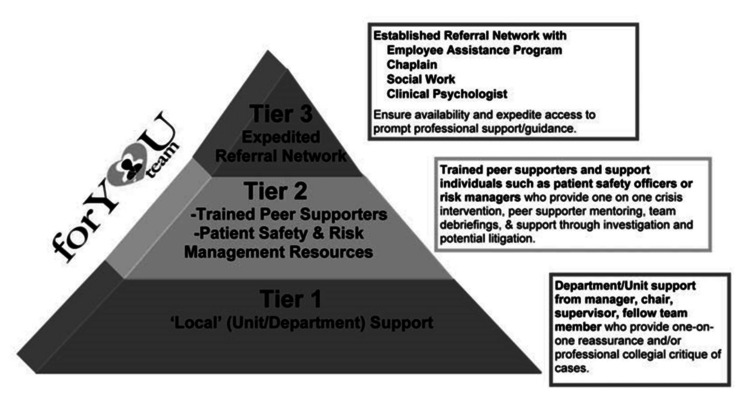
Scott's Three-Tiered Interventional Model of second victim support The Scott Three-Tiered Interventional Model of Support consists of three tiers, with the nature of support escalating from Tier 1 through Tier 3 Used with permission from Elsevier, from reference [24]

Many successful programs employ a three-tiered model, which includes basic emotional first aid provided by peers, immediate access to trained peer supporters (TPSs), and facilitated, destigmatized access to professional resources [18,20,23]. Scott’s Three-Tiered Model of Support (Figure 1) [24] served as the foundational framework for our program, guiding its design to ensure it was comprehensive and accessible.

Needs assessment

Before the implementation of our initiative, no formal peer support process existed in our anesthesia department. We conducted a comprehensive needs assessment using Scott's model [24], which emphasizes a tiered approach to addressing the needs of healthcare professionals after emotionally distressing events.

Based on observations and informal discussions with department members, this assessment revealed significant gaps in our support structure, specifically, the absence of Tier 1 (departmental support and psychological first aid) and Tier 2 (TPSs) interventions. Tier 3 services, such as Employee Assistance Programs (EAPs) and chaplains, exist but are commonly reported as inconvenient and underutilized due to stigma [20].

A custom survey was specifically developed for this project to assess the baseline level of perceived emotional support, awareness of SVP, and the support needs of anesthesia professionals within the department (see pre-implementation survey in the appendix). The survey incorporated elements from the Second Victim Experience and Support Tool (SVEST) [8] and included additional questions tailored to the unique context of our department. This survey was distributed electronically to all anesthesia professionals within the department, including CRNAs, physician anesthesiologists, and residents. The anonymous survey aimed to gather comprehensive insights into the current state of emotional support and identify key areas where peer support could be most beneficial.

Of 160 total anesthesia clinicians, 81 responded, yielding a response rate of approximately 50%. One in five respondents (16/82) reported experiencing an emotionally distressing clinical event in the preceding 30 days. Additionally, 88% (72/82) of respondents knew of a colleague emotionally affected by a work event, yet only 20% (16/82) felt these individuals had been adequately supported in the workplace. The results indicated over 90% of respondents (74/82) were likely to look to a trusted colleague for support after an emotionally challenging clinical event, and 65% (53/82) reported peer support as their most desired avenue. Findings were shared by the project lead with the department members and leadership at grand rounds. Key leaders and stakeholders fully endorsed the program development.

Program development

The toolkits from the Train-the-Trainer course through the Center for Patient Safety (led by Dr. S.D. Scott, creator of the forYOU program at the University of Missouri Health Care), the Betsy Lehmann Center for Patient Safety, and AMA STEPS Forward were particularly invaluable in the formation of our formal peer training workshop and materials [25-27]. These resources provide detailed guidance on structuring peer support programs and were instrumental in developing our training sessions’ evidence-informed content and practical format [24]. Consulting mentors from successful programs provided insights into best practices and challenges. These discussions, combined with the toolkit resources and evidence from the literature, influenced several key decisions in the design and rollout of our program, including the selection of peer supporters, the development of training materials, and the strategies used to promote awareness and engagement within the department.

To institute a team of formally TPSs, the project lead, a CRNA in the department with board certification in transformational wellness, tailored training materials, an agenda, and a team charter. Resources included a TPS workbook and a two-hour continuing medical education (CME)-approved presentation. The team, branded forU Peer Support, acquired an exclusive email address and a Teams page (Microsoft Corp., Redmond, WA, US) for operations. The name forU echoes Scott’s forYOU program, emphasizing individual well-being. At the same time, it subtly signals the program’s commitment to a broader team through the use of the “U,” which is a significant initial in our health system's logo.

Training

Our target was to have 5% of clinicians in the department complete the training, as recommended by Dr. Scott [26], to have an impactful and supportive presence. Department members nominated candidates based on suggested character traits outlined in the literature, such as being a good listener, trusted, caring, and strong in emotional intelligence [25-27]. Approximately 30 nominations were received, and emails were sent to coordinate the workshop date around the busy operating room schedule.

The first peer support workshop was executed in person less than two months after the completion of the needs assessment survey. The initial cadre of volunteer TPS team champions included six CRNAs, two physician anesthesiologists, and one resident anesthesiologist. The workshop received excellent reviews from all participants using a survey with Likert scales and open-ended questions, indicating improvements in knowledge and confidence in SVP support. After completing the training, participating clinicians signed a formal agreement of understanding, reinforcing confidentiality and TPS expectations.

forU Activation

The data collection period began on the first clinical workday following the training. TPS were readily available and open to discreetly meeting peers' emotional needs in person or via telephone. TPS wore forU-branded badges for identification and to stimulate discussion and awareness about the new program. Department members were notified of the peer support processes and resources via email, brochures, and bulletin boards. A private forU Teams page facilitated TPS communication and updates. Peer-to-peer encounters were initiated by proactive TPS outreach or requested in person by peers desiring support. Custom encounter forms to record data following each peer-to-peer support interaction were deidentified and completed anonymously by TPS using Microsoft Forms (Microsoft Corp., Redmond, WA, US), then collected and stored in a password-protected folder by the project lead (see encounter form in the appendix).

The anesthesiology department webmaster and project lead collaborated to add a peer support resource hub to the departmental intranet. Resources included a list of common events associated with SVP, possible signs and symptoms of SVP, coping and resilience strategies, emotional first-aid tips, a team roster, and contacts for professional mental health support. The hub was implemented on the departmental intranet during the second week of project implementation and was easily accessible to anyone. A department-wide notification was sent from the forU email to inform everyone about the new webpage. Due to scheduling conflicts, the project was not presented formally during grand rounds until the fifth week of implementation. At this time, the project lead gave a 20-minute presentation to the department describing the forU program efforts and progress to raise awareness and promote participation.

An additional training workshop was facilitated midway through the project period, increasing the number of TPS by 16 for a total of 15% of the department. Based on feedback from the previous group, all TPS received an innovative badge card with identification as a TPS, tips on facilitating the peer support process, and a professional mental health contact number.

The department chair authorized the team leader to access and screen the morbidity and mortality (M&M) reports before starting the project; however, the webmaster, M&M coordinator, and department administration did not facilitate sharing the reports with the project coordinator until the final data collection week. The support process currently includes a standardized outreach email from forU to any provider involved with an M&M report falling into the high-risk for second victim category; examples include unexpected patient death, preventable harm, an unanticipated event in a pediatric case, failure to rescue, and workplace violence events [18,20,21,23,26]. Applying a proactive approach is suggested in the literature and is crucial to the mission and sustainability of the project [18,20,21,23].

There are many interactive parts to this novel peer support process, most of which the project leader coordinates; information is shared with a co-leader to help eliminate gaps in team function. The encounter forms share open feedback offered by TPS, which is incorporated by the team into the improvement cycle. Promotional items (badges, infographics, and brochures), emails, verbal reminders, Teams page communication, and social media platforms are routinely utilized to raise awareness of the novel process.

Measures

Participation by anesthesia clinicians in the program was defined as the primary outcome metric and was measured by the number of deidentified peer support encounters reported and the number of times the online peer support resource hub was accessed. Process measures reflected data on peer support encounter forms, including a brief deidentified description of the event, length of the encounter, referral information, permission to follow up, and TPS self-evaluation of the peer support interaction. A key metric was the percentage of clinicians in the department who completed the formal peer support training to serve as TPS.

Balance measures used to gauge results included the average time spent providing and receiving support per department member and the TPS’s satisfaction rating (collected on the encounter form). Other balance measures included the percentage of respondents acknowledging perceived departmental emotional support and satisfaction ratings from those who received the formal support, collected via the department-wide electronic post-implementation survey (see post-implementation survey in the appendix).

Ethical considerations

Confidentiality and anonymity are crucial ethical concerns that all participants must ensure. As emphasized in signing the agreement of understanding, TPS maintained strict confidentiality regarding peer support encounters. Internal communications regarding peer support were strictly for departmental quality improvement (QI) and did not identify or detail participants, patients, or events in any way. This project received a waiver and was designated as QI by the Institutional Review Board (IRB).

## Results

**Figure 2:**
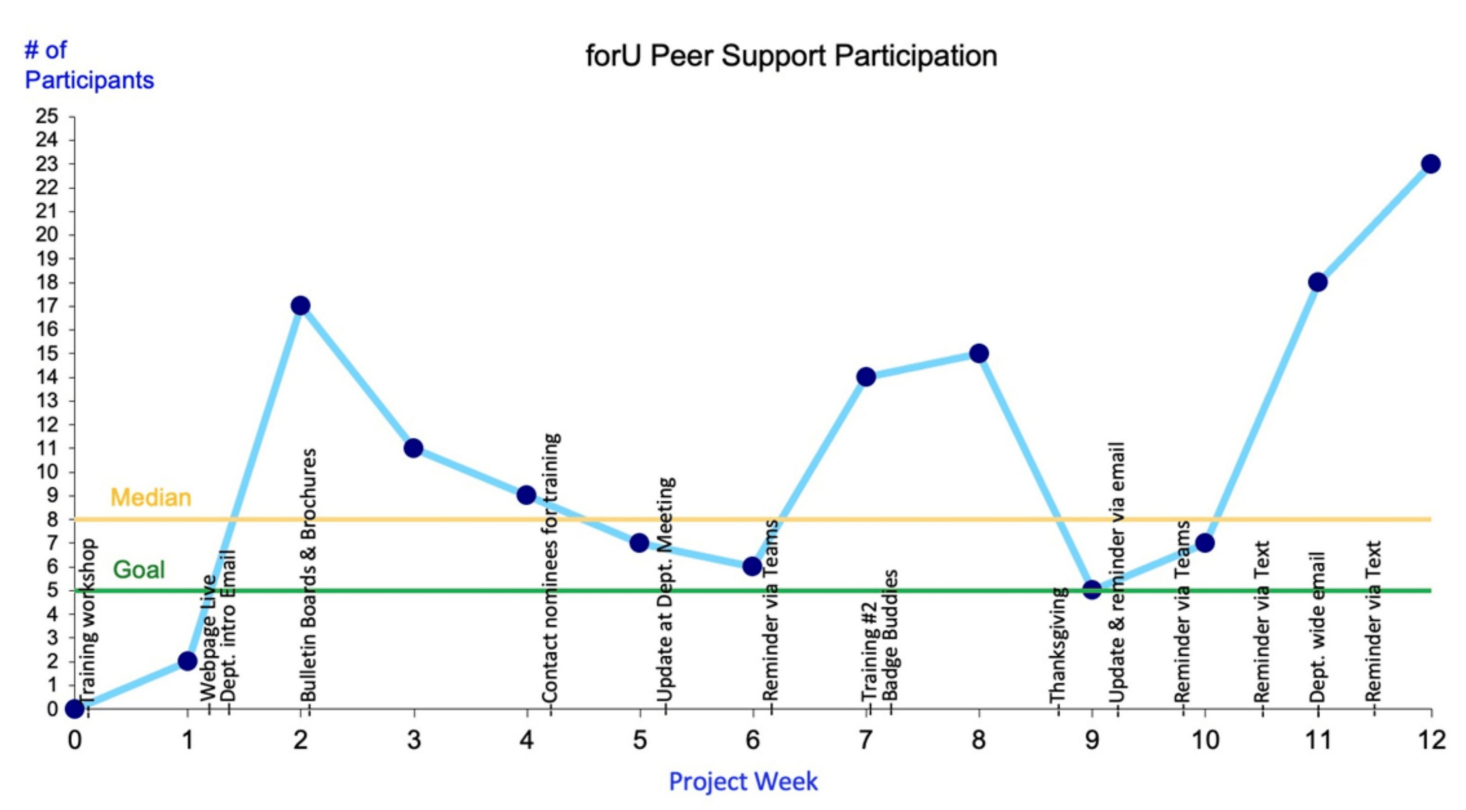
Run chart tracking the outcome measure of the QI project QI: quality improvement

**Figure 3:**
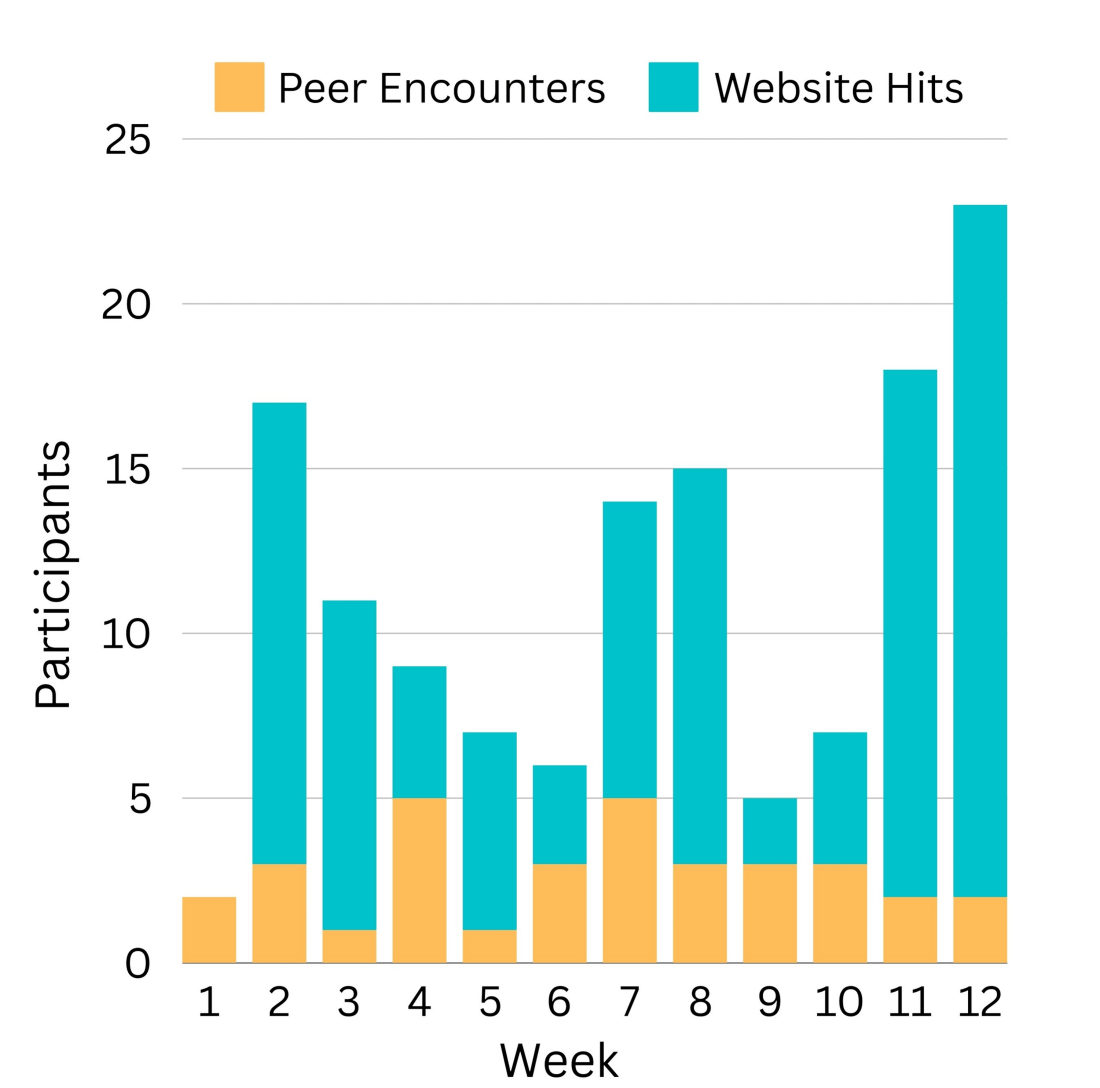
Outcome measure and participation breakdown

Participation was measured for 12 weeks post-implementation by the project lead. Figure 2 illustrates the number of participants (y) who utilized the process each week (x). The outcome measure was monitored weekly by totaling the deidentified peer support encounter forms recorded by TPS and the number of times clinicians accessed peer support resources on the website (participation breakdown shown in Figure 3). The run chart identifies strategic project interventions used to influence involvement over time.

Rises in participation correlate with the notations of reminders being sent and a second training workshop. The increased presence of TPS enhanced the visibility of the program and the accessibility of support. These timely improvements emphasize the importance of trend monitoring and departmental collaboration by a dedicated project lead. Participation decreased notably during the holiday week, likely due to reduced case scheduling and staffing, which may have resulted in fewer events warranting support.

TPS recorded 33 peer-to-peer encounters with an average time of 18 minutes, TPS satisfaction ratings of 4.6/5, and comfort with knowledge and skill as a supporter rating of 4.5/5. Peer support resources on the website were accessed more than 100 times in the first three months of availability and received an average rating of 4.7/5 from department members.

**Figure 4:**
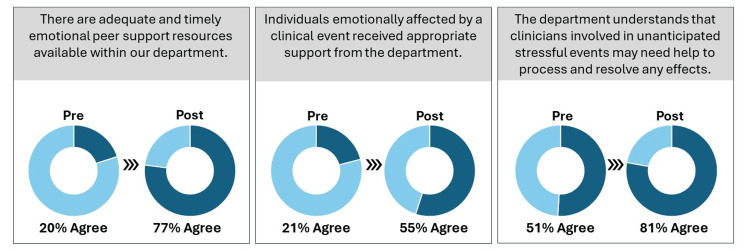
Comparison of pre-implementation and post-implementation perceived support

Figure 4 compares baseline and follow-up survey data from clinicians’ perceptions of support. A post-implementation survey using Likert scales and open-ended questions (response rate 61/175) was distributed electronically. It showed marked improvement in perceptions of emotional support within the department. Post-implementation survey results helped determine satisfaction with the process, the impact of interventions on perceptions of departmental support, and future directions. Familiarity with the term “second victim phenomenon” rose from 32% (26/81) to 66% (40/61) post-implementation.

Members of the anesthesia department praised the process improvement project in the open-ended feedback section of the survey. Satisfaction ratings by peers who received support from the forU program averaged 4.8/5. The open-ended section of the post-implementation survey included encouraging feedback such as “I reached out to a peer and had a very positive experience and felt supported”; “ForU person sought me out to help after an event. That was helpful”; “Very helpful, informative, and indeed a process improvement”; “Excellent support”; “Need help expanding to other services and providers”; and “I think many of us have trouble asking for help.”

## Discussion

Implementing the forU program demonstrated that a departmental peer support initiative could be established efficiently and effectively by peers, meeting the urgent need for emotional support among clinicians. The project was influential in building peer support resources to enhance workforce well-being and mitigate untoward outcomes from clinician distress. Participation exceeded five accounts most weeks, with high satisfaction ratings (see Figures 2, 3).

The project had a strong team champion presence, with 15% of clinicians being trained and identifiable by branded badges in the clinical setting. Collaborative engagement among multiple levels of anesthesia clinicians (physicians, CRNAs, and residents) facilitating and participating in forU is a testament to the importance of peer support and the success of the project.

**Figure 5:**
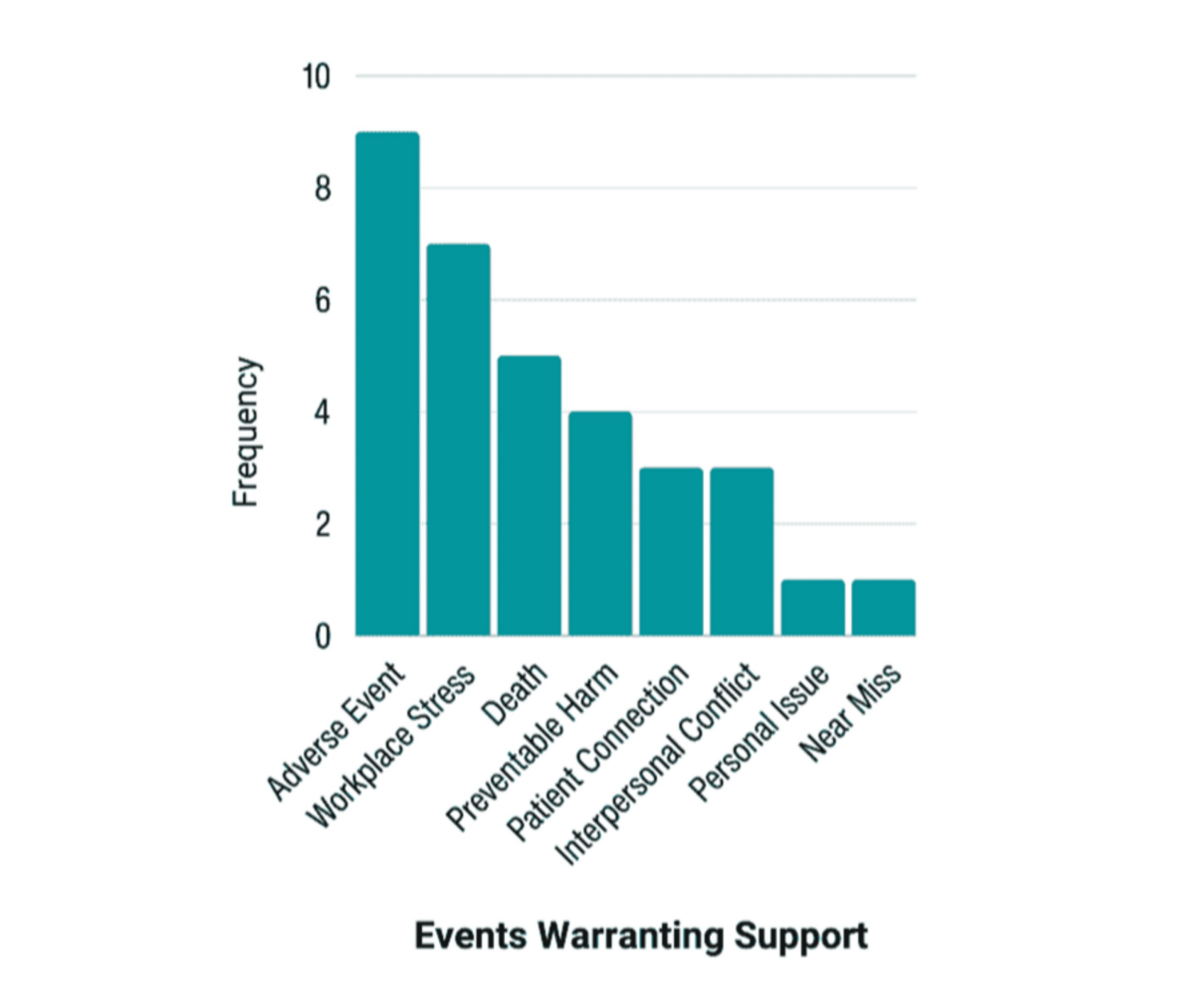
Type of event leading to TPS interaction TPS: trained peer supporter

No explicit details that could identify participants nor the incident precipitating event were recorded in the 33 support encounters. Many incidents warranting support were related to patient care and adverse events, but some were related to individual issues, such as work-life balance and workplace bullying. See Figure 5 for the categorization of events eliciting a peer support interaction.

Survey results showed that awareness of the term “second victim,” a critical preventative measure, doubled. The forU peer-to-peer support process is widely accessible and timely and aids in overcoming some of the barriers and stigmas of seeking help and support. Participants described the project as positive, effective, and helpful and encouraged its continuation and expansion to other disciplines within the health system.

Support for the project, voiced by leadership at departmental meetings, was vital to the success of initiating and implementing this innovative process. The Chair of Faculty Development offered full budgetary support for the program start-up from departmental funds. Its cost-effective nature was a critical factor in the success of the forU program. Expenses for the initiative were primarily limited to educational materials and promotional items. TPS participation was provided as in-kind support, with all contributions being voluntary and uncompensated. Each peer support encounter utilized fewer than 20 minutes per clinician involved, and the benefits likely outweighed the cost incurred in the absence of formal support. This model shows that departmental-level initiatives can be resource-efficient and highly impactful when executed methodically.

Moran and colleagues modeled a cost-benefit analysis of their peer support program, Resilience In Stressful Events (RISE), for nursing staff at Johns Hopkins [28]. They estimated a hospital cost savings of $22,576.05 per registered nurse who activated an encounter. Applying this estimate to the 33 forU encounters, the program could represent potential hospital savings of approximately $745,000. Furthermore, considering differences in pay and potential loss of revenue, the savings could be significantly higher when accounting for the participation of CRNAs and physicians compared to nurses.

Emotional peer support is associated with improved safety culture scores and individual and system resilience [18,19,21]. The benefits of peer support are far-reaching and can impact the clinicians participating in the process, the patients, and the entire health system. Research suggests that leadership support is essential for securing resources, promoting program visibility, and encouraging staff participation. Moreover, when leaders demonstrate a commitment to mental health and well-being initiatives, it signals to staff that these programs are valued, which can enhance engagement and reduce the stigma associated with seeking help [17]. Leadership involvement is also linked to better program sustainability and integrating peer support into the broader organizational culture, further reinforcing the importance of a supportive work environment [19,27]. Departmental support is a critical factor influencing a clinician’s decision to stay in the system after a challenging event [26]. Psychologically safe clinicians are likelier to maintain open communication and contribute to learning [29]. Leadership endorsement played a vital role in the seamless adoption of this program, highlighting how leadership support enhances engagement and fosters a culture of safety within the team.

This project's outcomes support Neft and associates' findings that formal peer support programs are the most effective and preferred method for supporting healthcare professionals who may be experiencing SVP [7]. While there are common themes of coping and recovery after emotionally challenging work events, each clinician has a unique experience and needs. The forU program is committed to engaging all three tiers of support: informal emotional aid by peers, TPSs, and destigmatized access to professional mental health resources [1,18,20,23]. Project leaders facilitated the expert recommendation of coordinating proactive support outreach with a standardized list of high-risk events [18,20,21,23].

Multiple team champions sustain activities and ensure that clinicians involved with critical events have increased awareness about the forU process. At orientation, a promotional infographic with information about the program and its resources is provided by leadership to new hires and residents. QR codes linking clinicians to online resources and raising awareness of the forU processes are displayed in anesthesia locations at all campuses. The forU team leaders are working with the Chief Wellness Officer and a Peer Support Advisory Committee to expand our program to other high-risk areas within the health system.

Strengths

A key strength of this initiative was its evidence-based foundation, integrating Havelock’s Model of Change and Scott’s Three-Tiered Model of Support, which provided a structured framework for implementation. The forU program was also strengthened by multidisciplinary involvement, engaging physician anesthesiologists, CRNAs, and residents in peer support efforts. Additionally, the initiative incorporated cost-effective and sustainable strategies, ensuring long-term viability with minimal resource burden.

Beyond its immediate impact, this model offers broader applicability to other clinical areas and healthcare settings, particularly in high-risk environments such as the operating room, emergency department, intensive care, obstetrics, and pediatrics. By fostering psychological safety, mitigating burnout, and enhancing clinician resilience, the forU program provides a replicable framework that aligns with national recommendations and organizational well-being initiatives.

Limitations

The pilot project encompassed a specialized group of clinicians in a singular academic health system. An advanced practice provider (APP), CRNA-Doctor of Nursing Practice (DNP), with board certification in holistic nurse coaching, spearheaded these efforts in partial fulfillment of doctoral scholarship. Healthcare leaders may face challenges replicating this process due to time constraints or competing priorities. Clinicians may underreport critical events and peer encounters due to stigma, time, and privacy concerns. While comparable to global findings, survey results are subject to responder bias. Although the initiative incorporated cost-effective strategies, ongoing funding, leadership engagement, and periodic training will be essential to maintaining its effectiveness. Integrating peer support into institutional policies could strengthen the long-term viability of similar initiatives across diverse healthcare settings. Future research should focus on evaluating the long-term impact of peer support initiatives on clinician retention, patient safety, and system resilience.

## Conclusions

This project aimed to develop and implement a peer support program to address anesthesia clinicians’ emotional and psychological needs. We identified critical gaps in our existing support structure and created a tailored, standardized, and accessible peer support program. The forU program effectively addressed the need for emotional and psychological support by increasing awareness of the SVP and promoting participation in peer support activities. The project aligns with broader healthcare objectives of fostering psychological safety and supportive work environments.

The incidence of challenging clinical events remains constant, highlighting the need for swift and urgent implementation of emotional support strategies. Sometimes, a safe space and a trusted confidant are needed to enable positive coping after an emotionally challenging event. This initiative showed that departmental programs can be efficient, cost-effective, replicable, and beneficial in high-stress patient care areas. By establishing support programs, like forU, healthcare systems can take a meaningful step toward prioritizing workforce well-being and fostering a culture of care.

## Figures and Tables

**Figure 6:**
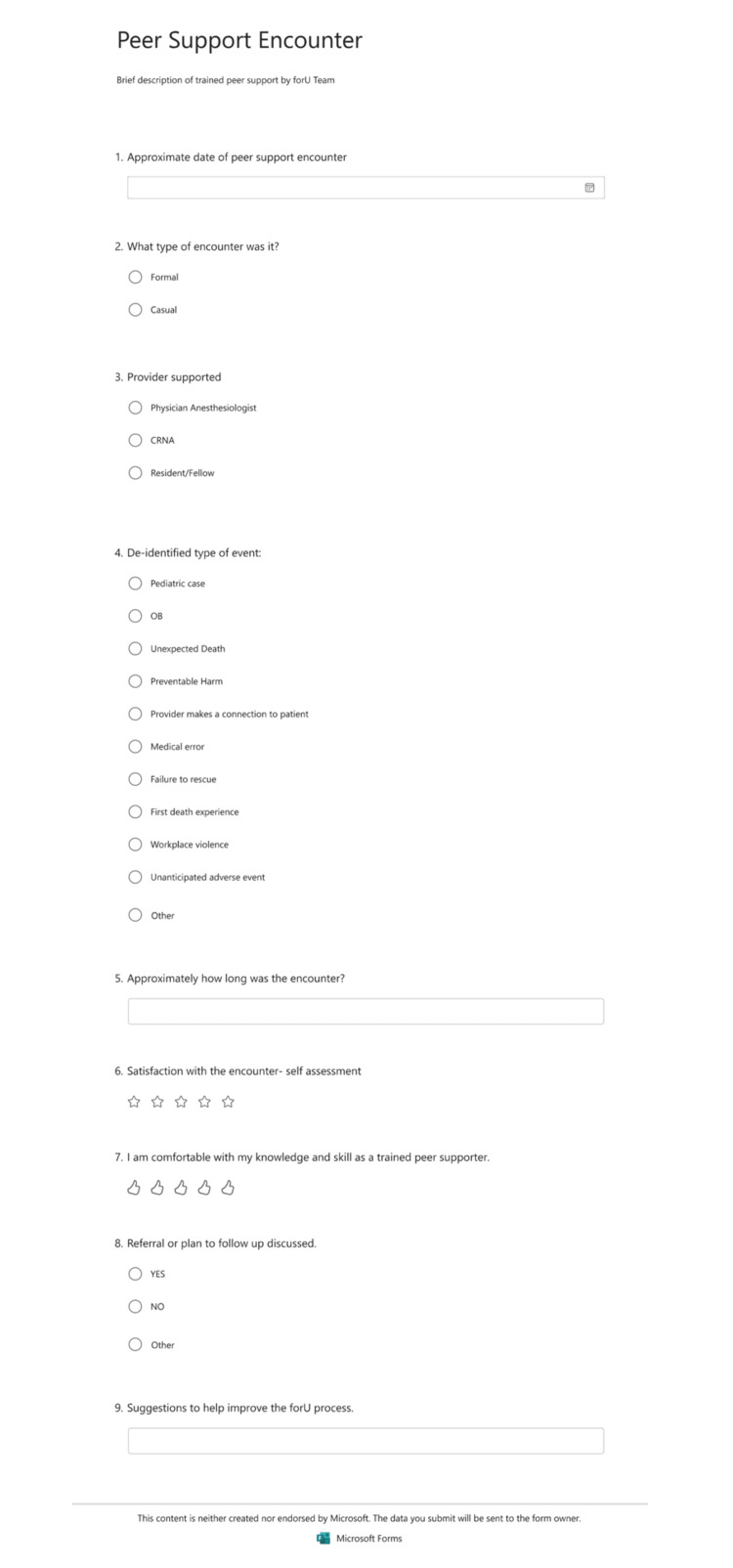
Encounter form

**Figure 7:**
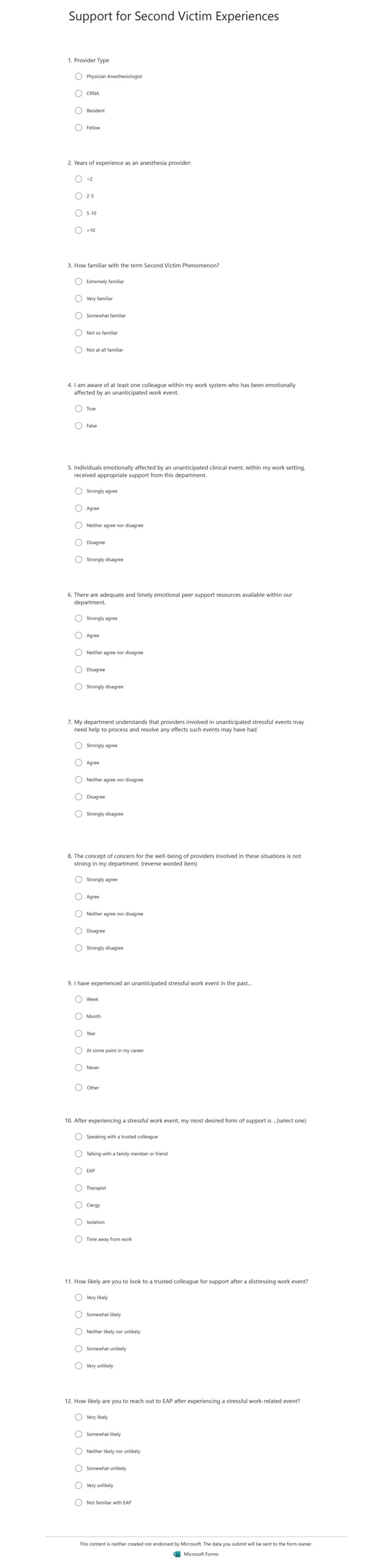
Pre-implementation survey

**Figure 8:**
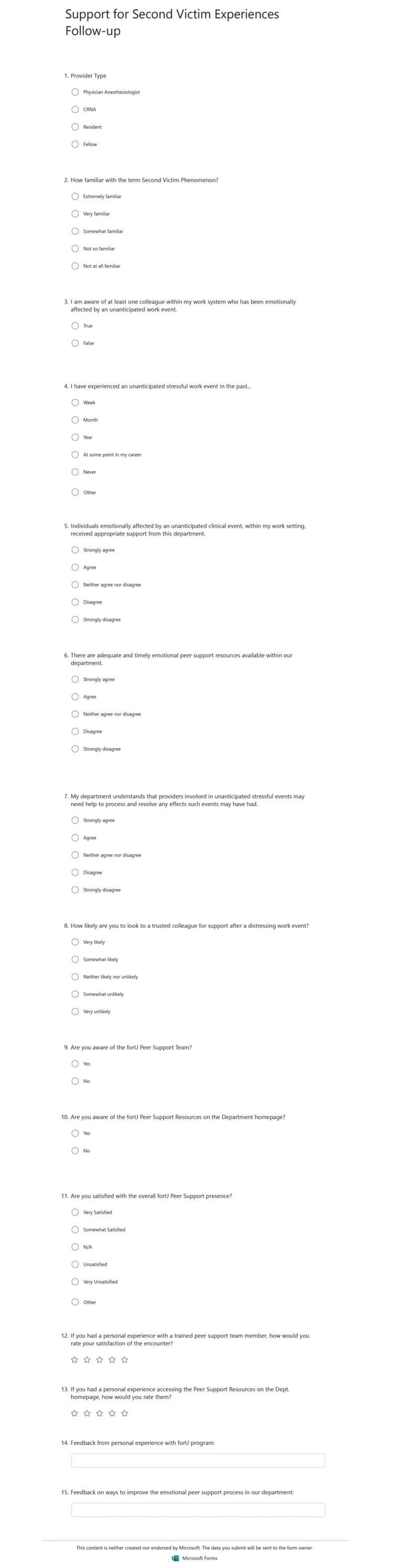
Post-implementation survey
